# Gastrotracheal Fistula as a Result of Transhiatal Esophagectomy for Esophageal Cancer: An Unusual Complication

**DOI:** 10.1155/2015/728393

**Published:** 2015-10-04

**Authors:** Heshmatollah Salahi, Mehdi Tahamtan, Bijan Ziaian, Mansoor Masjedi, Zahra Saadati, Nazanin Hoseini, Elahe Torabi

**Affiliations:** ^1^Transplant Research Center, Shiraz University of Medical Sciences, Shiraz, Iran; ^2^Colorectal Research Center, Shiraz University of Medical Sciences, Shiraz, Iran; ^3^Department of Thoracic Surgery, Shiraz University of Medical Sciences, Shiraz, Iran; ^4^Department of Anesthesiology, Shiraz University of Medical Sciences, Shiraz, Iran; ^5^Shiraz University of Medical Sciences, Shiraz, Iran; ^6^Department of Internal Medicine, Shiraz University of Medical Sciences, Shiraz, Iran

## Abstract

Gastrotracheal fistula following open transhiatal esophagectomy (Orringer's technique) for esophageal cancer is an unusual but lethal complication. Surgical intervention with resection of the fistula tract and primary interrupted suturing of gastric and tracheal orifices using a muscle flap interposition has proved to be a successful method. We report the case of a 73-year-old male with an adenocarcinoma of the distal part of the esophagus, who underwent open transhiatal esophagectomy (Orringer's technique) with gastric tube reconstruction and cervical anastomosis. The patient did not receive induction chemoradiotherapy before the esophagectomy. Two attempts of surgical repair of fistula failed and the patient died. Being aware of warning signs such as dyspnea and respiratory distress accompanied by bilious content in the tracheal tube is helpful in the early detection and treatment of this type of fistula.

## 1. Introduction

The development of gastrotracheal fistula after esophagectomy is a rare but life-threatening condition. Despite close anatomical relationship between the trachea and the stomach after esophagectomy, literature about gastrotracheal fistula is limited mainly to case reports. Diagnosis is based on both radiologic and endoscopic studies. The confirmation is often made by direct visualization of the fistula orifice by means of bronchoscopic or esophagoscopic modalities. Treatment options are conservative, endoscopic, and surgical, but the treatment of choice remains controversial.

## 2. Case Presentation

A 73-year-old male patient, who we knew to have had COPD and CABG, came to our clinic with uT3. No poorly differentiated adenocarcinoma at distal part of the esophagus was found. Endoscopy showed a 3.5 cm segment of narrowing in the lower third of the esophagus. He did not receive induction chemoradiotherapy before surgery.

The patient underwent an open transhiatal esophagectomy and reconstruction by gastric pull-up according to Orringer's technique.

Postoperative outcome was acceptable at first days and the patient was extubated successfully. On the 8th postoperative day, however, our patient developed dyspnea, cough, and respiratory distress. Intubation was therefore performed again. The patient underwent conservative management with supportive care and on the 30th postoperative day the patient was extubated successfully. During the next 6 days the patient developed respiratory distress with metabolic acidosis. Chest X-ray revealed bilateral basal consolidation with diffuse patchy infiltration. Consequently, the patient underwent intubation again. When the patient was reintubated, plenty large amount of bilious fluid was extracted from the endotracheal tube. Fiberoptic bronchoscopy was performed in the ICU and a fistula was detected 6 cm above the carina, just adjacent to the site of esophagogastric anastomosis. On the basis of bronchoscopic finding and clinical evidences, the diagnosis of gastrotracheal fistula was confirmed.

Because of the patient's bad general condition, conservative management and endoscopic approach for insertion of a stent in the trachea were not allowed. After a multidisciplinary team consultation, the patient was scheduled for operative intervention.

At the 1st operation, we carried out the left side neck exploration through the previous incision site. It showed severe inflammation and adhesion. A partial sternotomy was done to allow better access and exposure to the high retrotracheal portion of the esophagus [1]. After insertion of a nasogastric tube above the level of esophagogastric anastomosis, methylene blue was injected via NG tube to clarify the site of leakage. No leakage of dye appeared in the operating field. Then the gastric tube and cervical esophagus were mobilized and freed from fibrosis and adhesions. A fistula tract was identified in the posterior aspect of the anastomosis adjacent to the trachea. It was transected and repaired with interrupted primary sutures. Because the location of fistula was in the neck adjacent to the esophagogastric anastomosis, we could not interpose an intercostal muscle bundle, pericardial, pleural, or omental flap to protect the suture lines. Besides, the presence of severe inflammation and adhesions made interposition of strap muscle flap or sternocleidomastoid muscle (SCM) impossible. The patient was then transferred to the ICU.

**Figure 1:**
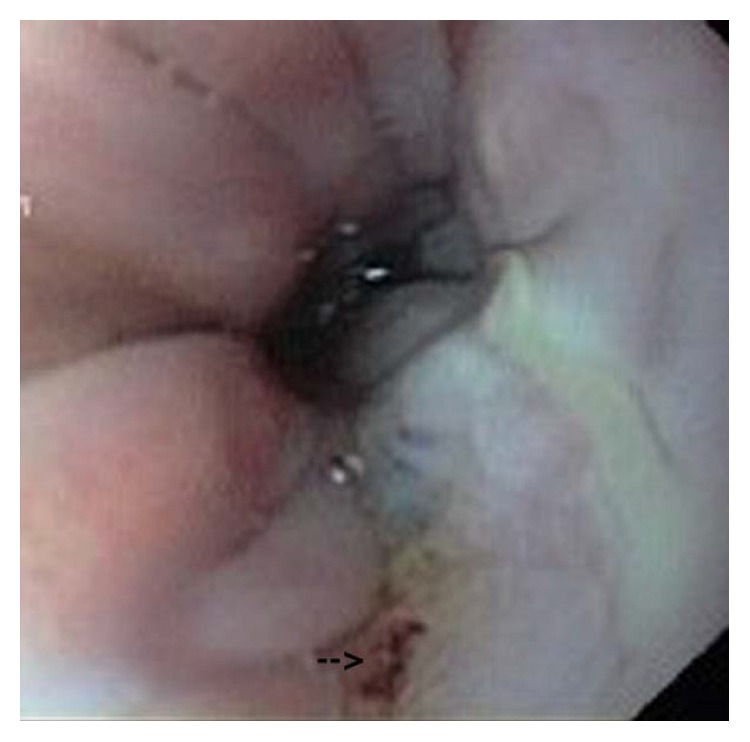
Gastrotracheal fistula just above the level of anastomosis.

After this operation, secretions of the endotracheal tube reduced dramatically but increased again at the 3rd postoperative day. Unfortunately, the subsequent endoscopy showed the persistence of the fistula about 16 cm from incisors, just above the level of anastomosis (Figure 1). The decision for the next neck exploration through the previous incision site was implemented 1 day later.

After exploration of the neck via previous incision, rigid esophagoscopy with transillumination of the esophagus was performed to detect the fistula orifice by one surgeon and at the same time gastrotracheal fistula and anastomosic leak was sutured by another surgeon. Besides, tracheostomy was done for the patient to eliminate the endotracheal tube cuff pressure on suture lines. As mentioned previously, interposing a muscle bundle was impossible.

The patient was then transferred to the ICU. In the ICU, the patient condition worsened and bilious secretions from the endotracheal tube continued. A new chest X-ray showed whitish lung which was in favor of chemical pneumonitis. Blood pressure and O_2_ saturation dropped and finally, the patient expired the next day after the second neck exploration.

## 3. Discussion

Literature on gastrotracheal fistula following esophagectomy for cancer consists mainly of case reports. This entity is rare (0.3–0.5%) but potentially lethal [1].

Several parameters contribute to selecting the best remedy such as general condition and severity of the disease and location and size of the fistula. In a patient with a good general condition, localized small fistula orifice, and no sign of necrosis, an endoscopic modality is the best option. In this method, abrasion-coagulation is followed by fibrin glue injection and, finally, approximation of the orifice with endoscopic clips [2]. As mentioned previously, if conservative management fails or if the patient's general condition worsens, a surgical intervention is imperative [3].

Surgical intervention consists of dissection of the fistula tract and repair of the esophageal and tracheal defects [4]. Additionally, the use of tissues with a rich blood supply (pleural, pericardial, myocutaneous, and muscle flaps) in the dead space between suture lines may protect the tracheal and gastroesophageal suture lines and prevent recurrent fistulization [5].

Symptoms vary widely from coughing to severe pneumonia, chemical pneumonitis, and mediastinitis [6]. There are various identified predisposing factors, the most important of which are previous chemoradiotherapy and en bloc resection with extensive lymphadenectomy [7].

But, what is the pathophysiology of fistula?

1. Leakage of the anastomosis causing mediastinal abscess formation and, in turn, secondary fistulization to the trachea [7].
2. Tracheal ischemia secondary to extensive dissection.
3. Iatrogenic tracheal injuries.
4. Cuff-induced tracheal ischemia secondary to prolonged intubation.
5. Tracheal injuries by gastric staplers.
6. Erosion of the stomach by tracheostomy tube.
7. Tumor recurrence.
8. Radiation.
9. Gastric ulcer [6].

One of the most important factors in preventing complications such as fistula is meticulous dissection of the tumors and careful esophagogastric anastomosis. Technical errors that must be avoided are tension on anastomosis, impairment of gastric or esophageal blood supply, mucosal defect of anastomosis, and overdistended gastric tube [5].

Gastrotracheal fistula after esophagectomy for cancers is a rare but life-threatening and challenging complication. It seems that the best choice in treating such kinds of fistula is surgical intervention, along with transposed pedicled pericardial, omental, pleural, or muscle flap. Even in a previously irradiated field, closure of the fistula with muscle bundle interposition such as SCM and pectoralis major myocutaneous (PMM) flap has showed excellent outcomes [8].

The SCM muscle flap for repair of an esophageal fistula and perforation is a well-known technique [9]. But in some reports there was a high rate of flap necrosis due to poor blood supply. So the pectoralis major muscle flap is the preferred method to repair the esophageal anastomotic leakage after gastric pull-up [10, 11]. However, the PMM flap is less flexible and requires a longer and difficult surgical procedure. In addition, cosmetic and functional complications at the donor site are more than those of the SCM flap [9]. PMM flap was not a good choice for our debilitated and asthenic patient. Most of the time, these two approaches are safe and feasible, especially when conservative management proves unsuccessful [3].

## 4. Conclusion

We report a case of gastrotracheal fistula after transhiatal esophagectomy (Orringer's technique) without a history of induction chemoradiotherapy. In our case, two attempts of gastrotracheal fistula repair with interrupted primary sutures without transposed flap were unsuccessful. If feasible, SCM muscle could help our patient. However, we refrained from SCM muscle flap because of the risk of flap necrosis and inability to interpose it in the appropriate place which had severely inflamed and adhesive tissue. We could not find an appropriate treatment for situations in which flap interposition is impossible. Being aware of warning signs such as dyspnea and respiratory distress accompanied by bilious content in the tracheal tube is helpful in the early detection and treatment of this type of fistula [12].
